# Wnt5a Increases the Glycolytic Rate and the Activity of the Pentose Phosphate Pathway in Cortical Neurons

**DOI:** 10.1155/2016/9839348

**Published:** 2016-09-05

**Authors:** Pedro Cisternas, Paulina Salazar, Carmen Silva-Álvarez, L. Felipe Barros, Nibaldo C. Inestrosa

**Affiliations:** ^1^CARE Biomedical Research Center, Faculty of Biological Sciences, Pontificia Universidad Católica de Chile, Alameda 340, P.O. Box 114-D, Santiago, Chile; ^2^Facultad de Ciencias Naturales, Departamento de Química y Biología, Universidad de Atacama, Copayapu 485, Copiapó, Chile; ^3^Centro de Estudios Científicos (CECs), Casilla 1469, Valdivia, Chile; ^4^Centre for Healthy Brain Ageing, School of Psychiatry, Faculty of Medicine, University of New South Wales, Sydney, NSW, Australia; ^5^Centro de Excelencia en Biomedicina de Magallanes (CEBIMA), Universidad de Magallanes, Punta Arenas, Chile

## Abstract

In the last few years, several reports have proposed that Wnt signaling is a general metabolic regulator, suggesting a role for this pathway in the control of metabolic flux. Wnt signaling is critical for several neuronal functions, but little is known about the correlation between this pathway and energy metabolism. The brain has a high demand for glucose, which is mainly used for energy production. Neurons use energy for highly specific processes that require a high energy level, such as maintaining the electrical potential and synthesizing neurotransmitters. Moreover, an important metabolic impairment has been described in all neurodegenerative disorders. Despite the key role of glucose metabolism in the brain, little is known about the cellular pathways involved in regulating this process. We report here that Wnt5a induces an increase in glucose uptake and glycolytic rate and an increase in the activity of the pentose phosphate pathway; the effects of Wnt5a require the intracellular generation of nitric oxide. Our data suggest that Wnt signaling stimulates neuronal glucose metabolism, an effect that could be important for the reported neuroprotective role of Wnt signaling in neurodegenerative disorders.

## 1. Introduction

Wnt ligands are critical for the correct function of the central nervous system. These molecules are implicated in several processes, including adult hippocampal neurogenesis, neuronal firing activity, the formation of the synaptic cleft, the enhancement of neuronal plasticity, and the regulation of mitochondrial dynamics [1, 2]. In this context, the ligand Wnt5a has been described as an important regulator of several neuronal processes, including protection against amyloid aggregation, dendritic spine formation, expression of microRNAs, and regulation of synaptic currents. Together, these results highlight the importance of Wnt5a in neuronal function [3–6]. Deregulation of Wnt signaling by either loss or gain of function is associated with the progression of various diseases, including cancer, diabetes mellitus type II, and neurodegenerative diseases, such as Parkinson's and Alzheimer's disease (AD) [7, 8]. In recent years, several reports have suggested a new role for Wnt signaling as a regulator of metabolic pathways [9, 10]. This idea has been proposed based on the indirect effects of Wnt signaling on other regulators/sensors of glucose metabolism, including phosphoinositide 3-kinase (PI3K) and AMP-activated protein kinase (AMPK), which are both involved in sensing the general metabolic state [11, 12].

In brain tissue, glucose is oxidized through glycolysis/oxidative phosphorylation to produce ATP, most of which is consumed by neurons during the restoration of ion gradients that are disrupted by synaptic transmission [13, 14]. Decreased glucose utilization by brain cells has been directly correlated with several brain pathologies, including AD [15, 16]. By contrast, enhancing glucose utilization* in vivo* induces significant improvements in cognitive functions, including memory and learning [17–19]. Despite the importance of glucose metabolism in brain function, no studies have described the effect of the Wnt5a pathway on glucose metabolism in cortical neurons.

In the present study, we demonstrate that Wnt5a stimulates glucose uptake, increases glycolytic rate, and stimulates the pentose phosphate pathway (PPP) in neurons. Additionally, Wnt5a treatment increases the activity of regulatory enzymes, such as hexokinase (HK) and glucose-6-phosphate dehydrogenase (G6PDH). The effect of Wnt5a was dependent on the production of nitric oxide (NO). These results suggest that the activation of Wnt signaling plays a central role in the regulation of neuronal metabolism and that this effect could be important for the correct function of the neuronal network.

## 2. Materials and Methods

### 2.1. Primary Neuronal Cell Cultures

Cortical neurons were obtained from the forebrains of 17-day-old rat embryos. The dissection was performed on samples immersed in dissection buffer containing 10 mM HEPES (pH 7.4, 320 mOsm/L). The tissues were incubated with 0.25% trypsin-0.2% EDTA (w/v) for 15 min at 37°C and then triturated to homogeneity with a fire-polished Pasteur pipette. The cells were seeded in poly-D-lysine-coated culture dishes at a density of 5*∗*10^5^ cells/cm^2^ and cultured in Dulbecco's modified Eagle's medium (DMEM) (Invitrogen, USA) containing 10% (v/v) fetal bovine serum (Thermo Fisher Scientific Inc., USA). After 30 min, the culture medium was changed to Neurobasal (NeuB) medium supplemented with B27 (Invitrogen, USA), 2 mM L-glutamine, 100 U/mL penicillin, 100 mg/mL streptomycin, and 2.5 mg/mL Fungizone (Thermo Fisher Scientific Inc., USA). The cell cultures were incubated in 5% CO_2_ in a humidified environment at 37°C. For all the experiments, the cells were used after 7 days* in vitro* [20, 21].

### 2.2. Generation of Control and Wnt5a-Conditioned Media

Control and Wnt5a-conditioned media were prepared from control L cells (American Type Culture Collection (ATCC): CRL-2648) and L-Wnt5a cells (ATCC: CRL­2814), respectively; the cells were cultured according to the protocol described by ATCC. Briefly, the cells were grown to 70% confluence in MEM supplemented with 10% FBS. The medium containing the Wnt ligand was recovered (batch 1), and this process was repeated with the same cells after an additional 4 days in culture (batch 2). Then, batches 1 and 2 of the conditioned media were combined [20, 22–26].

### 2.3. Cell Treatment

The neurons were cultured in NeuB supplemented with B27 until the day of the experiments. Before the treatment with the Wnt ligand, the cells were maintained in NeuB without B27 for 1 h (to avoid the possible effects of the growth factors present in B27). Then, the cells were incubated with control or Wnt5a media (1-2 h). The neurons were also treated with inhibitors of Wnt signaling; the inhibitors were coincubated with the control media or Wnt5a media. The inhibitors used were FzCRD-2 (5 ng/mL, antagonist of Wnt ligands), KN-93 (10 *μ*M, inhibitor of CaM kinase II), Gö6976 (200 nM, inhibitor of PKC), and 7-nitroindazole (7-NI; 5 *μ*M, inhibitor of nitric oxide synthases). All the Wnt inhibitors were obtained from Sigma-Aldrich, USA.

The neurons were also treated with inhibitors of glucose metabolism, such as cytochalasin B (Cyt B, 20 *μ*M, inhibitor of glucose transporters (GLUTs)), cytochalasin E (Cyt E, 20 *μ*M, control of the action of Cyt B) [27], 2-deoxy-D-glucose (2-DG, 7 mM, competitive inhibitor of HK) [28], and dichloroacetate (DCA, 5 mM, inhibitor of glycolysis that blocks the activity of the pyruvate dehydrogenase complex) [29]. Moreover, we treated the neurons with sodium nitroprusside (SNP, 0–800 nM), a NO donor. In all cases, the neurons were treated with the inhibitors for 30 min prior to the experiment with or without the Wnt5a media.

### 2.4. Glucose Uptake Analysis

After the treatment with Wnt5a in the presence or absence of Wnt inhibitors, the neurons were carefully selected using a microscope to ensure that only plates containing cultures of uniformly growing neurons were used. Following incubation with the Wnt ligand, the cells were washed with incubation buffer (15 mM HEPES, 135 mM NaCl, 5 mM KCl, 1.8 mM CaCl_2_, and 0.8 mM MgCl_2_) supplemented with 0.5 mM glucose [30]. Uptake was measured at room temperature by the addition of 1–1.2 *μ*Ci of 2-deoxy-D-[1,2-(N)^3^H] glucose ([2-^3^H]-DG) at a final specific activity of 1–3 disintegrations/min/pmol (approximately 1 mCi/mmol). Uptake was stopped by washing the cells with ice-cold PBS supplemented with 1 mM HgCl_2_. The incorporated radioactivity was assayed by liquid scintillation counting. For the pharmacological experiments, the cells were incubated with the glucose metabolism inhibitors for 30 min in the presence of a radioactive substrate. For the slice experiments, the slices were incubated with ([2-^3^H]-DG) for a specific period, and then a standard methodology was used. The kinetic parameters were determined using a single rectangular hyperbola of the form *V*_max_ *∗*[glc]/(*K*_*m*_ + [glc]), which was adjusted to the data by nonlinear regression using SigmaPlot 12 [31]. The ([2-^3^H]-DG) was purchased from PerkinElmer, USA.

### 2.5. Quantification of Hexokinase (HK) Activity

After treatment with the Wnt ligand, the neurons were washed with PBS, treated with trypsin/EDTA, and centrifuged at 500 ×g for 5 min at 4°C. Then, the cells were resuspended at a 1 : 3 dilution in isolation medium (250 mM sucrose, 20 mM HEPES, 10 mM KCl, 1.5 mM MgCl_2_, 1 mM EDTA, 1 mM DTT, 2 mg/mL aprotinin, 1 mg/mL pepstatin A, and 2 mg/mL leupeptin), sonicated at 4°C, and then centrifuged at 1,500 ×g for 5 min at 4°C. Finally, the HK activity of the supernatant was quantified. For the assay, the purified fraction was mixed with the reaction medium (25 mM Tris-HCl, 1 mM DTT, 0.5 mM NADP/Na^+^, 2 mM MgCl_2_, 1 mM ATP, 2 U/mL G6PDH, and 10 mM glucose) and incubated for 30 min at 37°C. The reaction was stopped by the addition of 10% trichloroacetic acid (TCA), and NADPH production was measured at 340 nm [32].

### 2.6. Determination of the Glycolytic Rate

The glycolytic rates were determined using previously described methods [30, 33]. After treatment with Wnt5a, the cells were detached from the culture plates using trypsin/EDTA (Sigma-Aldrich, USA). Next, the neurons were placed in tubes containing 5 mM glucose and then washed twice in Krebs Henseleit solution (11 mM Na_2_HPO_4_, 122 mM NaCl, 3.1 mM KCl, 0.4 mM KH_2_PO_4_, 1.2 mM MgSO_4_, and 1.3 mM CaCl_2_, pH 7.4), containing the appropriate concentration of glucose. After equilibration in 0.5 mL of Hank's balanced salt solution/glucose at 37°C for 10 min, 0.5 mL of Hank's balanced salt solution containing various concentrations of [3-^3^H] glucose was added, with a final specific activity of 1–3 disintegrations/min/pmol (approximately 1 mCi/mmol). Aliquots of 100 *μ*L were then transferred to another tube, placed inside a capped scintillation vial containing 0.5 mL of water, and incubated at 45°C for 48 h. After this vapor-phase equilibration step, the tube was removed from the vial, a scintillation mixture was added, and the ^3^H_2_O content was measured by scintillation counting over a 5 min period. The [3-^3^H] glucose was obtained from PerkinElmer, USA. The cell viability after the experiment was approximately 90%.

### 2.7. Determination of Glucose-6-Phosphate Dehydrogenase (G6PDH) Activity

After treatment with the respective compound, the cells were washed with PBS, collected by scraping in 0.25% trypsin-0.2% EDTA (w/v), and pelleted. Subsequently, the pellet was discarded, and the supernatant was further separated by centrifugation at 13,000 ×g for 30 min at 4°C. Finally, the supernatant was used to quantify the G6PDH activity in a reaction buffer consisting of 1 mM ATP and 10 mM glucose-6-phosphate (G6P) after a 30-min incubation at 37°C. The reaction was stopped by the addition of 10% TCA, and the NADPH production was measured at 340 nm [32].

### 2.8. Measurement of Glucose Oxidation through the Pentose Phosphate Pathway (PPP)

Glucose oxidation via the PPP was measured using a previously described method [34], which is based on the difference in ^14^CO_2_ production from [1-^14^C] glucose (decarboxylated in the 6-phosphogluconate dehydrogenase-catalyzed reaction and in the Krebs cycle) and [6-^14^C] glucose (only decarboxylated in the Krebs cycle). After the treatment with Wnt5a in the presence or absence of inhibitors, the medium was removed, and the neurons were washed with ice-cold PBS and collected by trypsinization. The cell pellets were resuspended in O_2_-saturated Krebs Henseleit buffer (11 mM Na_2_HPO_4_, 122 mM NaCl, 3.1 mM KCl, 0.4 mM KH_2_PO_4_, 1.2 mM MgSO_4_, and 1.3 mM CaCl_2_, pH 7.4), and 500 *μ*L of this suspension (~10^6^ cells) was placed in Erlenmeyer flasks with 0.5 mL of the Krebs Henseleit solution containing 0.5 *μ*Ci D-[1-^14^C] glucose or 2 *μ*Ci D-[6-^14^C] glucose and 5.5 mM D-glucose (final concentration). The Erlenmeyer flasks were equipped with a central well containing an Eppendorf tube with 500 *μ*L of benzethonium hydroxide. The flasks were flushed with O_2_ for 20 s, sealed with rubber caps, and incubated for 60 min in a 37°C water bath with shaking. The incubations were stopped by the injection of 0.2 mL of 1.75 M HClO_4_ into the main well, although shaking was continued for an additional 20 min to facilitate ^14^CO_2_ trapping by benzethonium hydroxide. Radioactivity was assayed by liquid scintillation spectrometry [35, 36]. Both [1-^14^C] glucose and [6-^14^C] glucose were purchased from PerkinElmer, USA.

### 2.9. ATP Content

After the treatment with Wnt5a, we measured the ATP levels in whole-cell lysates of primary neurons using an ATP determination kit (Invitrogen/Molecular Probes) [37].

### 2.10. Animals and Ethical Standards

Slices were prepared from 2-month-old male C57BL/6 mice. The animals were housed at the Animal House Facility of the Facultad de Ciencias Biológicas, Pontificia Universidad Católica de Chile, in accordance with the Guide for the Care and Use of Laboratory Animals (NIH-USA Publication 86-23).

### 2.11. Slice Preparation

Hippocampal slices were prepared from 60-day-old C57BL/6L mice using standard procedures. Transverse slices (350 *μ*m) from the dorsal hippocampus were cut in cold artificial cerebrospinal fluid (ACSF, 119 mM NaCl, 26.2 mM NaHCO_3_, 2.5 mM KCl, 1 mM NaH_2_PO_4_, 1.3 mM MgCl_2_, and 10 mM glucose) using a Vibroslice microtome (World Precision Instruments) and incubated in ACSF for 1 h at room temperature. The experiments were performed in a recording chamber at room temperature (20–22°C) [38, 39].

### 2.12. Statistical Analysis

All the experiments were performed with an *n* of 5; we used triplicates for each condition of each experiment. The results are expressed as the mean ± standard error. The data were analyzed by one-way or two-way analysis of variance (ANOVA) followed by Bonferroni's post hoc test; ^*∗*^ *p* ≤ 0.05 and ^*∗∗*^ *p* ≤ 0.01 were considered significant. Statistical analyses were performed using Prism software (GraphPad, USA).

## 3. Results

### 3.1. Activation of Wnt Signaling Stimulates Glucose Uptake in Neurons

**Figure 1:**
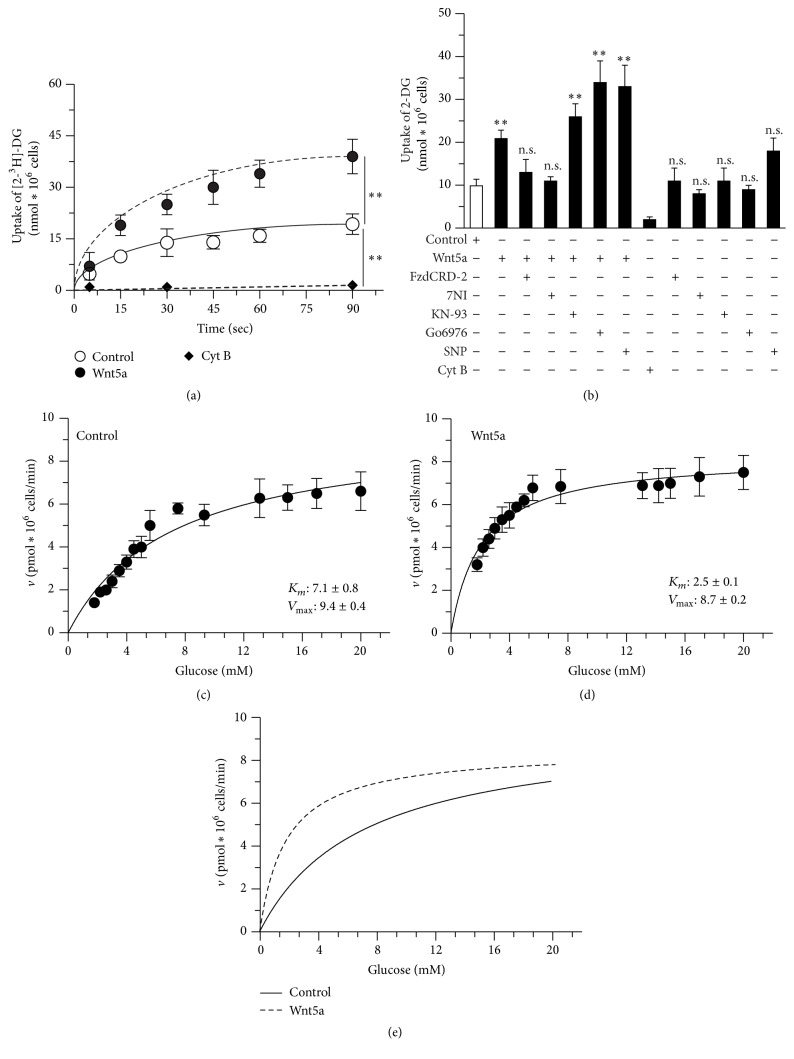
Wnt5a stimulates glucose uptake. (a) Treatment with Wnt5a stimulates the uptake of 2-DG in a time-dependent manner. (b) The effect of Wnt5a was blocked by the antagonist FzCRD2 and with 7NI a nNOs inhibitor. (c–e) The initial uptake of tracer amounts of 2-DG (at 15 sec.) was measured in the presence of increasing concentrations of unlabeled glucose (0–30 mM), under the control condition (c) and in cells treated with Wnt5a (d). Representative plot of both conditions (e). Data represent the mean ± SEM of *n* = 4, each performed in triplicate. ^*∗∗*^ *p* < 0.005, Bonferroni test.

The activation of the noncanonical Wnt pathway was determined by monitoring the p-PKC levels. We observed a 1.5-fold increase in the p-PKC/PKC ratio after 2 h of Wnt5a treatment, and this increase was blocked by the Wnt scavenger FzCRD-2 (data not shown). The first step of glucose metabolism is the uptake of glucose from the extracellular media into the cell. We used 2-deoxyglucose (2-DG), a radioactive analog of glucose, to study glucose uptake. In the brain, glucose is taken up by glucose transporters (GLUTs) and phosphorylated by the enzyme HK, but it is not further metabolized [40]. Under control conditions, we observed a time-dependent uptake of 2-DG, with a maximum of 19.2 ± 3.5 nmol *∗*10^6^ cells at 90 sec. After the Wnt5a treatment, the 2-DG uptake increased, with a maximum of 39.4 ± 5.3 nmol *∗*10^6^ cells at 90 sec. Wnt pathway activation triggered a 2-fold increase in 2-DG uptake, with a significant difference at 15 sec. Treatment with the GLUT inhibitor Cyt B (20 *μ*M) decreased 2-DG uptake to 1.56 ± 0.4 nmol *∗*10^6^ cells at 90 sec (Figure 1(a)).

Incubation with Wnt5a during the initial phase of monitoring induced an increase in uptake from 9.9 ± 1.5 nmol *∗*10^6^ cells to 20.8 ± 2.0 nmol *∗*10^6^ cells; this increase was blocked by the coincubation with FzCRD-2. The treatment with this inhibitor alone did not alter the 2-DG uptake.

Interestingly, we observed that 7-NI, an inhibitor of neuronal NO synthase (nNOS) [41, 42], blocked the effect of Wnt5a, suggesting that the effect of this ligand was mediated by the intracellular release of Ca^2+^ followed by the downstream generation of NO. We treated the cells with inhibitors of several downstream targets of noncanonical Wnt signaling to further investigate this effect. Neither KN-93 (inhibitor of calcium/calmodulin-dependent protein kinase II (CaMKII)) nor Gö6976 (inhibitor of PKC) blocked the effect of Wnt5a. By contrast, treatment with the NO donor SNP alone increased the 2-DG uptake to 17.8 ± 4.3 nmol *∗*10^6^ cells (Figure 1(b)).

The kinetic parameters of glucose uptake were estimated for a more comprehensive analysis of the effect of Wnt signaling. Under control conditions, we observed a *K*_*m*_ value of 7.1 ± 0.8 mM and a *V*_max_ of 9.4 ± 0.4 pmol*∗*10^6^ cells/min (Figure 1(c)). In the Wnt5a-treated cells, the *K*_*m*_ and *V*_max_ values were 2.5 ± 0.1 mM and 8.7 ± 0.2 pmol*∗*10^6^ cells/min, respectively (Figure 1(d)).  Figure 1(e) illustrates how exposure to the Wnt5a ligand increased the apparent affinity of glucose in the neuronal transport pathway. By contrast, there was no apparent change in the *V*_max_ for transport.

### 3.2. Activation of Wnt Signaling Increases the Glycolytic Rate of Cortical Neurons

**Figure 2:**
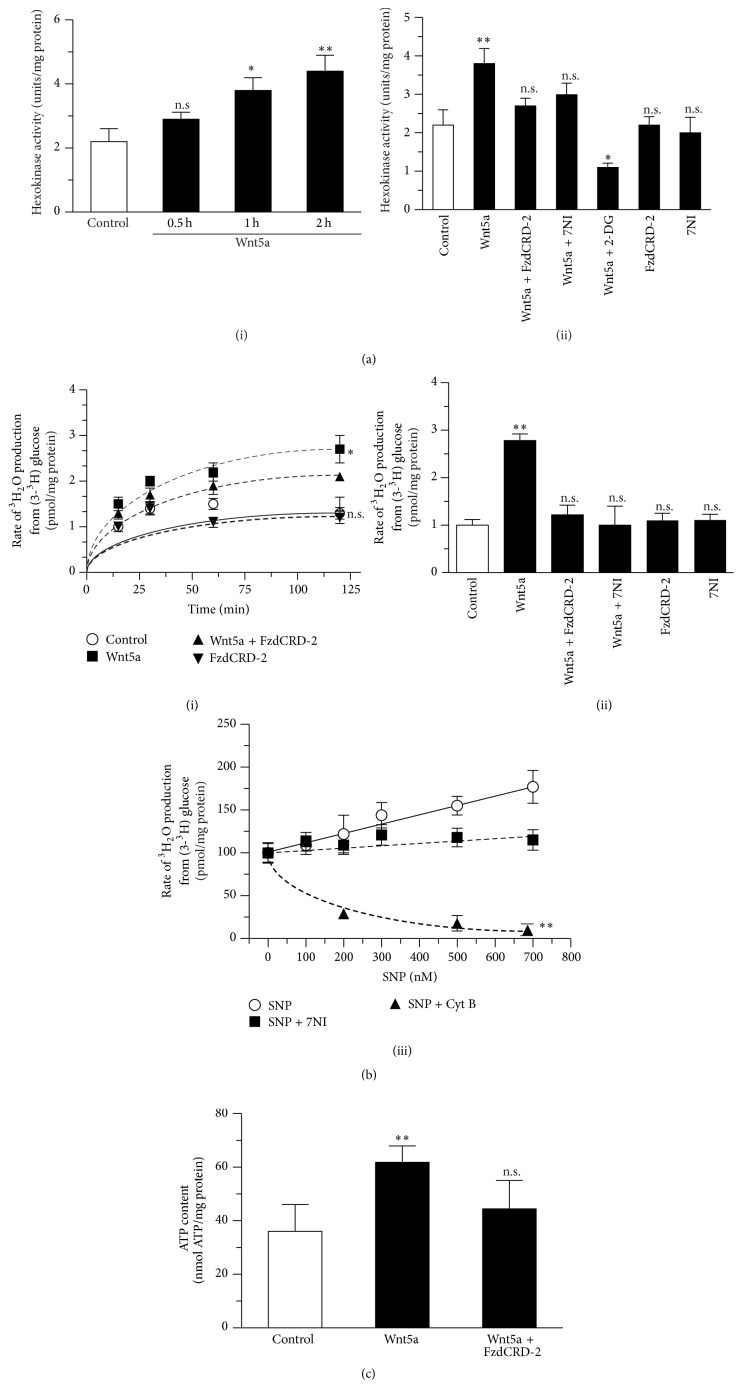
The activation of noncanonical Wnt pathways stimulates the glycolytic rate in cortical neurons. (a) Treatment with Wnt5a stimulates the HK activity in a time-dependent manner (i), and this effect was blocked by coincubation with FzdCRD-2 and 7NI (ii). (b) Incubation with Wnt5a stimulates the glycolytic rate (i); this effect was blocked by coincubation with 7NI and FzCDR-2 and stimulated in a concentration-dependent manner by the NO donor SNP ((ii) and (iii)). (c) Treatment with Wnt5a by 2 h increases ATP production; this effect was blocked by the coincubation with FzdCRD-2. Data represent the mean ± SEM of *n* = 6, each performed in triplicate. ^*∗*^ *p* < 0.01; ^*∗∗*^ *p* < 0.005, Bonferroni test.

After being transported into the cell, glucose is converted to G6P by HK, the first regulatory enzyme of glycolysis [43]. Thus, we studied the effect of activation of Wnt5a signaling on HK activity. We observed that the Wnt5a treatment induced a robust increase in activity from 2.2 ± 0.17 units/mg to 4.4 ± 0.8 units/mg after 2 h of treatment (Figure 2(a), (i)). The effect of Wnt5a was prevented by the coincubation with FzCRD-2 and 7-NI (Figure 2(a), (ii)).

Our results thus far show that treating neurons with Wnt5a stimulates the uptake of 2-DG and HK activity. The next step was to determine whether this increased uptake was correlated with an increase in the glucose utilization through glycolysis. We used radiolabeled [3-^3^H] glucose to test this possibility [33]. First, we measured the glycolytic rate at several time points after treatment with Wnt5a. Under control conditions, we observed rates of 1.1 ± 0.15 pmol/mg protein. Wnt5a increased the glycolytic rate in a time-dependent manner, with a maximum rate of 2.9 ± 0.3 pmol/mg protein at 2 h versus a control rate of 1.3 ± 0.19 pmol/mg protein. This effect was partially blocked by the coincubation with FzCRD-2 (Figure 2(b), (i)). Using a pharmacological approach, we studied the effects of several inhibitors at 2 h and observed that the effect of Wnt5a was blocked by the coincubation with FzCRD-2 and 7-NI (Figure 2(b), (ii)).

Our previous results suggest that the downstream generation of NO following the activation of noncanonical Wnt signaling may be important for the effect of Wnt5a on the glycolytic rate. Therefore, we analyzed the effect of SNP, a NO donor, by treating cells with several concentrations of SNP for 2 h; we observed a strong increase in the glycolytic rate, with a maximum of 150 ± 12 pmol/mg protein in the cells that were treated with 700 nM SNP. The effect of SNP was partially abolished by the coincubation with 7-NI. As a control, the Cyt B treatment markedly decreased the glycolytic rate in neurons (Figure 2(b), (iii)).

Our results indicate that the activation of Wnt signaling stimulates glucose uptake and the utilization of this molecule by glycolysis, suggesting an increase in the levels of ATP. We measured the neuronal ATP levels following a 2 h treatment with Wnt5a to confirm this prediction. Under control conditions, we measured a value of 36.02 ± 10.03 nmol ATP/mg of protein. After the treatment with Wnt5a, the ATP levels increased to 61.78 ± 6.11 nmol ATP/mg of protein; this increase in the ATP levels was blocked when cells were coincubated with Wnt5a and the antagonist FzdCRD-2 (Figure 2(c)).

### 3.3. Activation of Wnt Signaling Stimulates PPP Activity

**Figure 3:**
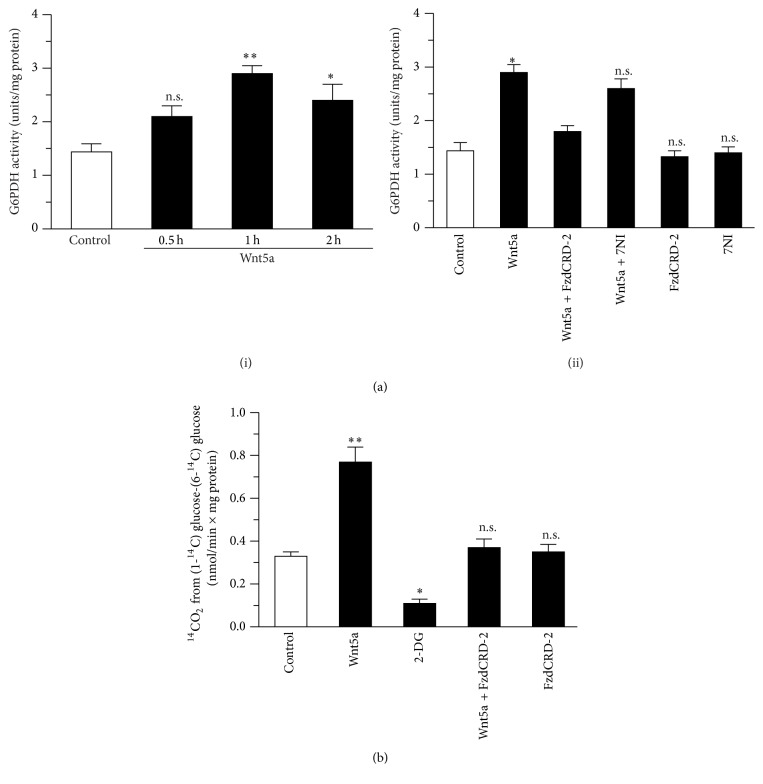
The Wnt5a stimulates the PPP activity. (a) The activation of Wnt pathways increases the activity of G6PDH in a time-dependent manner (i); this effect was blocked by the coincubation with FzdCRD-2 and 7NI (ii). (b) The oxidation of glucose through PPP pathway was stimulated after the treatment with Wnt5a; this effect was blocked by the coincubation with FzdCRD-2. Data represent the mean ± SEM of *n* = 5, each performed in triplicate. ^*∗*^ *p* < 0.01; ^*∗∗*^ *p* < 0.005, Bonferroni test.

As described above, several metabolic pathways could use the G6P generated by HK, including the PPP. The PPP is critical for the reduction of NADP^+^ to NADPH. We measured the effect of Wnt5a on G6PDH activity and observed an increase in the enzymatic activity after 1 h of Wnt5a treatment (Figure 3(a), (i)). The effect of Wnt5a on G6PDH activity was blocked by the coincubation with FzCRD-2 and 7-NI (Figure 3(a), (ii)). Then, we measured the PPP activity using radioactive glucose and observed that the Wnt5a treatment increased the PPP activity from 0.33 ± 0.02 nmol/min × mg protein in the control condition to 0.77 ± 0.07 nmol/min × mg protein. The effect of Wnt5a on the PPP activity was blocked by the coincubation with FzdCRD-2 (Figure 3(b)).

### 3.4. The Wnt5a Ligand Stimulates Glucose Utilization in Hippocampal Slices

**Figure 4:**
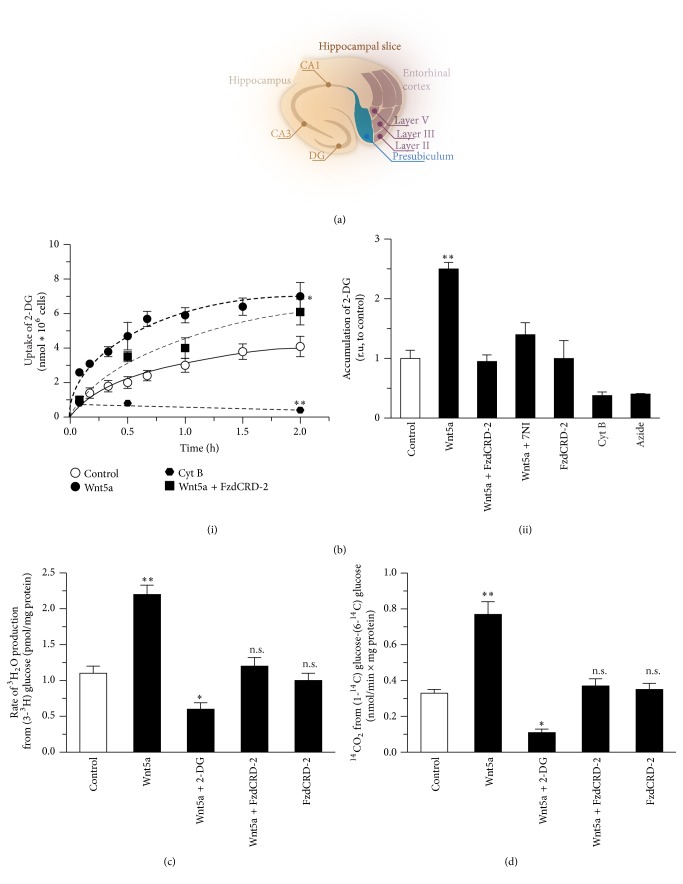
Wnt pathway activation stimulates the glucose metabolism in hippocampal slices. (a) Schematic representation of 350 *μ*m hippocampal slice. (b) In slices, Wnt5a treatment induced an increase in the intracellular accumulation of 2-DG in a time-dependent manner (i); this effect was blocked by the coincubation with FzdCRD-2 (ii). (c) Slices incubated with Wnt5a showed an increase in the glycolytic rate; this effect was blocked by 2-DG and FzCRD-2. (d) In slices we observed an increase in the PPP activity. Data represent the mean ± SEM of *n* = 5, each performed in triplicate. ^*∗*^ *p* < 0.01; ^*∗∗*^ *p* < 0.005, Bonferroni test.

We studied whether the effects of the Wnt5a ligand could be recapitulated in a more complex model to further analyze the effect of Wnt pathway activation on glucose metabolism. For this experiment, we used cortical-hippocampal slices from mouse brains (Figure 4(a)). After a 30 min treatment, we observed significant time-dependent differences in the accumulation of 2-DG, with a maximum of 6.9 ± 0.9 nmol *∗*10^6^ after 2 h of Wnt5a treatment compared with 3.4 ± 0.4 nmol *∗*10^6^ for the control condition (Figure 4(b), (i)). The effect of a 1 h Wnt5a treatment was blocked by treatments with FzdCRD-2 and 7-NI in a similar manner to that observed in neuronal cultures (Figure 4(b), (ii)). After the 1 h incubation with Wnt5a, we observed an increase in the glycolytic rate in the slices from 1.07 ± 0.11 pmol/mg protein to 2.12 ± 0.08 pmol/mg protein; this effect was blocked by the coincubation with 2-DG and FzdCRD-2 (Figure 4(c)). Finally, we measured the PPP activity in slices and observed an activity of 0.32 ± 0.01 nmol/min × mg protein in the controls. The Wnt5a treatment induced an increase in the PPP activity to 0.78 ± 0.02 01 nmol/min × mg protein; this effect was blocked by the coincubation with FzdCRD-2 and 7-NI (Figure 4(d)).

## 4. Discussion

In the present work, we studied the effect of Wnt5a on glucose metabolism in cortical neurons. We report that Wnt5a treatment stimulates glucose uptake in a time-dependent manner; this increase was correlated with an increase in both the HK activity and the glycolytic rate. Moreover, the treatment with Wnt5a increased the activity of the G6PDH enzyme and the PPP. Both processes depend on the generation of NO downstream of the Wnt5 signaling. Together, these results suggest that the activation of Wnt signaling by Wnt5a regulates cellular glucose metabolism in neurons.

Wnt signaling can basically be divided into two pathways: the canonical pathway (Wnt/*β*-catenin) and the noncanonical pathway [44]. Wnt5a has been described as a noncanonical Wnt ligand, and the noncanonical pathway includes the planar cell polarity (Wnt/PCP) and Wnt/Ca^2+^ pathways. In the Wnt/PCP pathway, the Wnt ligand binds to the Fzd receptor and activates small GTPases that subsequently induce the expression of genes related to the reorganization of the cytoskeleton. In the Wnt/Ca^2+^ pathway, ligand binding to the Fzd receptor activates the enzyme phospholipase C (PLC), which increases the production of inositol triphosphate (IP_3_), generating an increase in the intracellular Ca^2+^ concentration that leads to the activation of calcium-dependent proteins [45–47]. Subsequently, Wnt5a signaling increases the intracellular levels of several second messengers, including Ca^2+^ and NO, which are both considered regulators of glucose metabolism, a critical process required for brain function [48–51].

The importance of the Wnt pathway in regulating glucose metabolism has been increasingly recognized in recent years due to studies in humans, where several components of the Wnt pathway have been identified as risk factors for metabolic diseases, including age-related dementia; however, the final effect depends on whether the canonical or noncanonical Wnt pathway is affected [52, 53]. Activation of the Wnt/*β*-catenin pathway promotes a decrease in the plasma glucose levels* in vivo*, and this decrease modulates the localization and expression of GLUT4 in adipocytes and increases glucose uptake in these cells [54]. Similarly, patients with diabetes mellitus type II have been shown to exhibit increased expression of sFRP5, a Wnt inhibitor, which has been correlated with a decrease in Wnt5a levels [55]. Furthermore, activation of the Wnt/Ca^2+^ pathway has been reported to modulate mitochondrial dynamics, which may affect ATP generation [56, 57].

Brain tissue exhibits a high rate of glucose utilization. Approximately 20% of the oxygen and 25% of the glucose consumed by the human body are dedicated to cerebral functions, even though the brain only accounts for 2% of the total body mass [58]. The ATP generated by glucose oxidation is used to maintain and restore the ion gradients dissipated by signaling processes, such as postsynaptic stimulation and action potentials, and for neurotransmitter uptake and recycling [13]. Glucose is the main energy substrate of the adult brain, and its metabolism is mainly divided into two stages: glucose uptake and intracellular oxidative metabolism [59].

We observed that the Wnt5 treatment stimulates glucose uptake in cortical neurons. Our data also showed that Wnt5a increases the apparent affinity of the GLUT3 transporter, the main GLUT isoform expressed in neurons [60]. Because we did not observe changes in the *V*_max_ value, we can disregard the expression of other GLUT isoforms in our experiments. The effect of Wnt5a on glucose uptake will increase the bioavailability of intracellular glucose, with a subsequent increase in the G6P levels generated by HK, another target of the Wnt5a pathway. The generated G6P is mainly used by three different metabolic pathways: glycolysis, the PPP, and the glycogen synthesis pathway.

**Figure 5:**
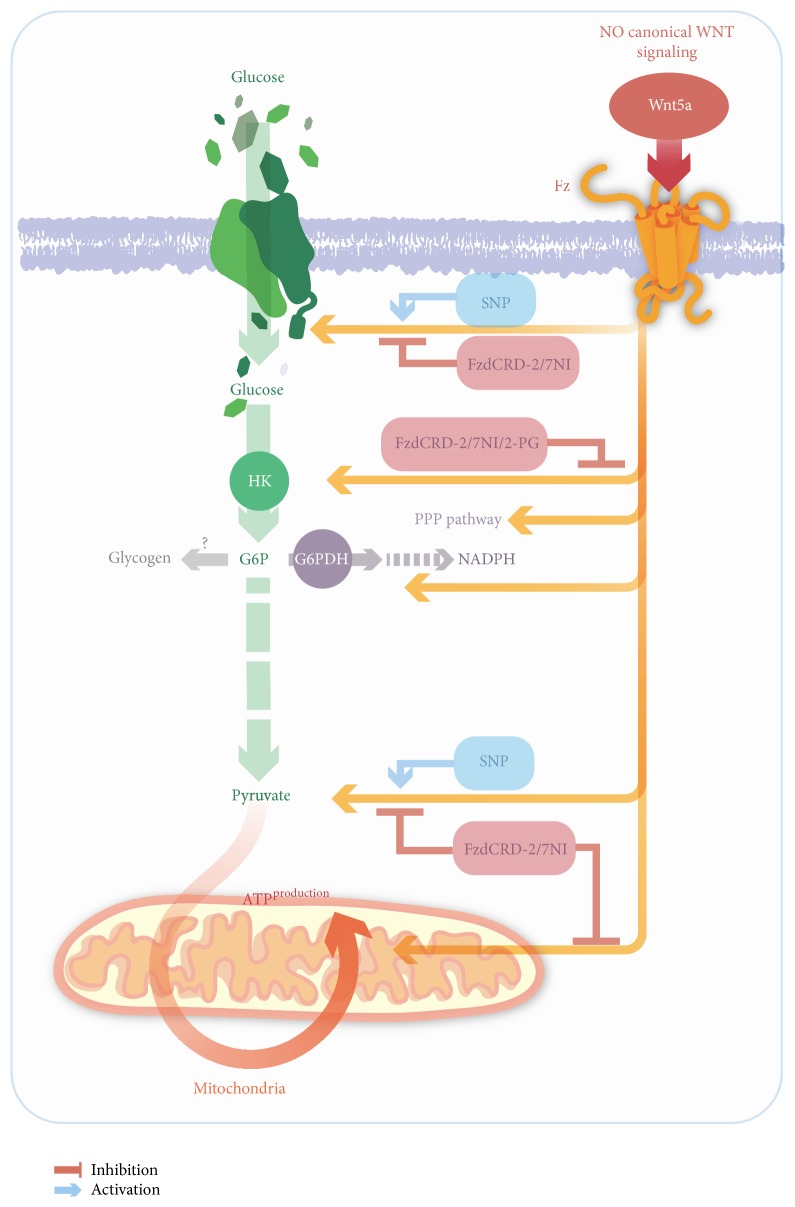
Schematic representation of the effect of Wnt5a over the glucose metabolism in cortical neurons. The Wnt5 stimulates the glucose uptake and the activity of the HK. Downstream the Wnt5a stimulates the glycolytic rate and PPP activity, in a NO-dependent manner. The increase in the glycolytic rate was correlated with an increase in the generation of ATP. The increase in the PPP activity (through the production of the cofactor NADPH) could be correlated as a mechanism against the oxidative stress generated by the increase in the mitochondria activity.

Glycolysis is coupled to the Krebs cycle and oxidative phosphorylation to generate substrates for ATP production in mitochondria through oxidative phosphorylation. By contrast, the PPP is required to protect neurons from oxidative stress through the generation of NADPH [61, 62]. However, the neuronal utilization of glucose for glycogen synthesis has been accepted for years [63]; neurons have recently been shown to use glucose for glycogen synthesis, a function that is typically attributed to astrocytes. However, this glycogen synthesis could induce neuronal cell death through the formation of glycogen aggregates called Lafora bodies [64, 65]. Neuronal glycogen may be important for promoting neuronal survival under pathological conditions, such as oxidative stress and hypoxia [66–68]. In the present work, we did not evaluate the effect of Wnt5a on glycogen synthesis, but we cannot discard this possibility because the importance of glycogen in neurons has recently been reported [67, 69]. We observed that Wnt5a increases the glycolytic rate and PPP activity, thus promoting ATP generation and increasing the levels of the NADPH cofactor. The increase in the NADPH levels resulting from PPP activity could be a protective mechanism against oxidative stress (generated by the increased ROS levels produced in the mitochondria), because this molecule is critical for the recycling of antioxidant agents, such as ascorbic acid and glutathione [69, 70]. Additionally, we observed that the effects of Wnt5a on 2-DG uptake and the glycolytic rate were blocked by an inhibitor of nNOS. This finding suggests a role for intracellular NO, a molecule generated downstream of the Wnt/Ca^2+^ pathway, because Ca^2+^ release activates calcineurin, a phosphatase that leads to nNOS activation and NO production (Figure 5) [41, 71]. NO has been previously shown to stimulate glucose metabolism in neurons; however, the specific mechanism underlying this effect remains unknown [72, 73].

Because the brain includes other cells, such as astrocytes, which play critical roles in the regulation of neuronal metabolism [74–76], we tested the effects of Wnt5a on the more complex system of hippocampal slices. Here, we observed results similar to those of the* in vitro* studies, suggesting that the effects of Wnt5a are mainly localized to neurons; however, further studies are required to confirm this hypothesis.

## 5. Conclusions

Our results suggest a novel function for Wnt signaling as an activator of glucose metabolism in neurons. This novel role of Wnt signaling in neuronal physiology might be an interesting topic in the search for new treatments for neurological disorders.
